# American Dental Association

**Published:** 1897-09

**Authors:** 


### Reports of Society Meetings.

### AMERICAN DENTAL ASSOCIATION.

THE Thirty-seventh Annual Session of the American Dental Association was opened in the winter ball-room of the Hotel Chamberlain, at Old Point Comfort, on Tuesday, August 3, 1897, at eleven o'clock, A.M., the President, Dr. James Truman occupying the chair.

On motion, the calling of the roll and reading of the minutes was dispensed with.

The Secretary, Dr. Cushing, read the resignation of Dr. Morris L. Chaim, which was accepted.

The report of the Publication Committee, read by Dr. Cushing, showed that the transactions were printed in the usual manner and distributed among the members, libraries, etc., about seventy copies being still on hand. The report also presented an account of the money expended by such committee.

Referred to the Finance Committee.

### REPORT OF THE EXECUTIVE COMMITTEE.

*Dr. Crouse.*—As chairman of the Executive Committee, I sent to all the members two communications. I sent out a list of subjects early in the spring, with the request to take them up and discuss them, and give us a synopsis or digest of their work for the year. Later, I sent another communication giving railroad rates, and urging attendance at this meeting. During the spring I came here to look after the arrangements, and decided upon this hotel as being the best one for us. I chose this room for the meetings, with the understanding that if it were not satisfactory it could be changed. This large meeting-room and the smaller committee rooms are given to us by the hotel free of charge, and I was enabled to make a rate of $2.50 per day.

I also made railroad arrangements for the different roads, except the Western Passenger Road, which would not give us any rates. The programme presented is the one arranged for the meeting, although we can make changes in it from time to time as is considered best. I will present the bills for expenses later.

Report received and approved as far as given.

The treasurer's report, by Dr. Henry W. Morgan, was as follows:

| | |
|---|---|
| Balance on hand at last report | $1294.28 |
| Collected at Saratoga, and since | 1080.00 |
| Total | 2374.28 |
| Expended | 768.94 |
| Leaving on hand | $1605.34 |

The expenditures were as follows:

| | |
|---|---|
| Salary of the Secretary for 1896 | $200.00 |
| Salary of the Treasurer for 1896 | 100.00 |
| Reporter for session of 1896 | 125.00 |
| Printing, expressage, and postage | 134.20 |
| On account of National Museum | 53.95 |
| Executive Committee expenses of the Saratoga meeting | 78.59 |
| Publication Committee | 40.00 |
| Expenses of Section I. | 15.00 |
| Expenses of Section II. | 11.20 |
| Expenses of Section IV. | 6.00 |
| Refunded dues, one member | 5.00 |
| Total | $768.94 |

Report referred to the usual committee.

COMMITTEE ON HORACE WELLS MEMORIAL.

*Dr. Cushing.*—I have a communication from Dr. J. D. Thomas, of Philadelphia, giving a statement of account of the Horace Wells Memorial Fund, as follows:

| | | |
|---|---|---|
| Amount of subscriptions to date | | $1057.73 |
| Expenses | $68.01 | |
| Unpaid subscriptions, A. D. A. | 250.00 | |
| Unpaid subscription, Richmond Dental Society | 50.00 | |
| Unpaid subscription, individuals | 12.50 | |
| | | 380.51 |
| Amount in bank | | $677.22 |

Report placed on file and referred to Publication Committee.

COMMITTEE ON STATE AND LOCAL SOCIETIES.

*Dr. Crouse.*—I tried to get a closer relation between the local societies and this society. How successful I have been will be seen later. As to Section III., Operative Dentistry, I sent to the secretary of that section a card to be mailed to all members of the American Dental Association, asking them to bring samples of the amalgam they were using in their practice, so we might make tests here with the micrometer, dynamometer, and other scientific means. We will have a clinic for that purpose, which will take a day or two probably.

COMMITTEE ON NATIONAL MUSEUM.

*Dr. Donnelly.*—We have not been able to meet, so we ask for further time to make our report.

Request granted.

The Committee on Necrology also asked for further time, which was granted.

The Committee on Violation of the Code of Ethics, through Dr. Jackson, reported progress, and asked permission to present a fuller report later in the session.

The Vice-President, Dr. Thomas Fillebrown, then took the chair and the President read his address.

(For this address, see page 563.)

Dr. Truman, having met with a slight accident, the discussion on his report was deferred until the evening session, and the meeting adjourned until half-past seven.

(To be continued.)

